# Supplementary material for: Changing Decisions: The Interaction between Framing and Decoy Effects
Source: Behav Sci (Basel). 2023 Sep 12;13(9):755. doi: 10.3390/bs13090755 (PMC10525293; doi:10.3390/bs13090755)
Supplement: Supplementary file 1 [file behavsci-13-00755-s001.zip › behavsci-2555034-supplementary.pdf]

Changing decisions: the interaction between Framing and Decoy effects

Supplementary Materials

Table S1. Chi-square analysis for males and females.

**Gender**	**Condition**	**Risk-averse choice**	**Risk-seeking choice**	χ^2	**p**
Male	Original positive	64.20%	35.80%	39.620	<.001
	Congruent decoy positive	82.80%	17.20%	205.270	<.001
	Incongruent decoy positive	53.20%	46.80%	1.765	.18
	Original negative	26.40%	73.60%	109.387	<.001
	Congruent decoy negative	24.10%	75.90%	115.648	<.001
	Incongruent decoy negative	41.60%	58.40%	13.406	<.001
Female	Original positive	72.3%	27.7%	276.692	<.001
	Congruent decoy positive	78.1%	21.9%	429.864	<.001
	Incongruent decoy positive	54.5%	45.5%	9.744	<.001
	Original negative	27.6%	72.4%	279.179	<.001
	Congruent decoy negative	24.0%	76.0%	329.077	<.001
	Incongruent decoy negative	49.9%	50.1%	.005	.94

Table S2. Chi-square analysis for each scenario included in the study.

**Scenario**	**Condition**	**Risk-averse choice**	**Risk-seeking choice**	χ^2	**p**
Crew	Original positive	69.4%	30.6%	48.024	<.001
	Congruent decoy positive	75.2%	24.8%	77.475	<.001
	Incongruent decoy positive	53.8%	46.2%	1.496	.220
	Original negative	26.9%	73.1%	68.089	<.001
	Congruent decoy negative	22.7%	77.3%	83.771	<.001
	Incongruent decoy negative	43.6%	56.4%	4.866	.027
Fatal disease	Original positive	70.9%	29.1%	55.038	<.001
	Congruent decoy positive	82.5%	17.5%	140.810	<.001
	Incongruent decoy positive	55.5%	44.5%	3.086	.079
	Original negative	34.2%	65.8%	31.455	<.001
	Congruent decoy negative	28.0%	72.0%	50.917	<.001
	Incongruent decoy negative	48.4%	51.6%	0.312	.576
Pregnancy	Original positive	75.3%	24.7%	80.651	<.001
	Congruent decoy positive	75.5%	24.5%	78.290	<.001
	Incongruent decoy positive	59.7%	40.3%	10.049	.002

	Original negative	20.9%	79.1%	106.698	<.001
	Congruent decoy negative	22.6%	77.4%	81.983	<.001
	Incongruent decoy negative	43.5%	56.5%	4.952	.026
Money	Original positive	73.8%	26.3%	71.974	<.001
	Congruent decoy positive	83.6%	16.4%	136.830	<.001
	Incongruent decoy positive	61.4%	38.6%	14.504	<.001
	Original negative	28.8%	71.3%	57.619	<.001
	Congruent decoy negative	22.0%	78.0%	88.122	<.001
	Incongruent decoy negative	52.0%	48.0%	0.485	.486
Cab	Original positive	68.0%	32.0%	39.528	<.001
	Congruent decoy positive	77.9%	22.1%	95.589	<.001
	Incongruent decoy positive	48.2%	51.8%	0.364	.550
	Original negative	22.9%	77.1%	89.598	<.001
	Congruent decoy negative	28.9%	71.1%	47.905	<.001
	Incongruent decoy negative	55.3%	44.7%	3.360	.067
Homeselling	Original positive	63.4%	36.6%	21.906	<.001
	Congruent decoy positive	81.4%	18.6%	122.653	<.001
	Incongruent decoy positive	46.9%	53.1%	1.111	.291
	Original negative	30.1%	69.9%	48.313	<.001

Congruent decoy negative	20.1%	79.9%	99.056	<.001
Incongruent decoy negative	43.3%	56.7%	5.369	.020

Figure S1. Frequency of choice of the decoy option.

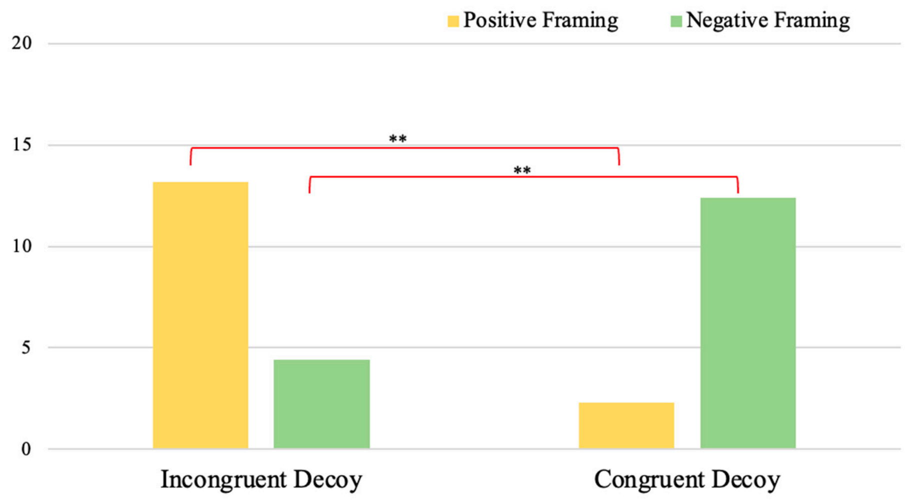

Note. ** $p < .001$. Only the significant comparisons that have been discussed in the Results section of the manuscript are represented.
